# Clinical Research Informatics: a Decade-in-Review

**DOI:** 10.1055/s-0044-1800732

**Published:** 2025-04-08

**Authors:** Christel Daniel, Peter J. Embí

**Affiliations:** 1 AP-HP, France; 2 Sorbonne Université, INSERM UMR_S 1142, LIMICS, F-75006, Paris, France; 3 Vanderbilt University Medical Center, Department of Biomedical Informatics, Nashville, Tennessee, USA

**Keywords:** Clinical Research Informatics, Biomedical Research, Clinical Trials, Informatics, Literature Review

## Abstract

**Background** : Clinical Research Informatics (CRI) is a subspeciality of biomedical informatics that has substantially matured during the last decade. Advances in CRI have transformed the way clinical research is conducted. In recent years, there has been growing interest in CRI, as reflected by a vast and expanding scientific literature focused on the topic. The main objectives of this review are: 1) to provide an overview of the evolving definition and scope of this biomedical informatics subspecialty over the past 10 years; 2) to highlight major contributions to the field during the past decade; and 3) to provide insights about more recent CRI research trends and perspectives.

**Methods** : We adopted a modified thematic review approach focused on understanding the evolution and current status of the CRI field based on literature sources identified through two complementary review processes (AMIA CRI year-in-review/IMIA Yearbook of Medical Informatics) conducted annually during the last decade.

**Results** : More than 1,500 potentially relevant publications were considered, and 205 sources were included in the final review. The review identified key publications defining the scope of CRI and/or capturing its evolution over time as illustrated by impactful tools and methods in different categories of CRI focus. The review also revealed current topics of interest in CRI and prevailing research trends.

**Conclusion** : This scoping review provides an overview of a decade of research in CRI, highlighting major changes in the core CRI discoveries as well as increasingly impactful methods and tools that have bridged the principles-to-practice gap. Practical CRI solutions as well as examples of CRI-enabled large-scale, multi-organizational and/or multi-national research projects demonstrate the maturity of the field. Despite the progress demonstrated, some topics remain challenging, highlighting the need for ongoing CRI development and research, including the need of more rigorous evaluations of CRI solutions and further formalization and maturation of CRI services and capabilities across the research enterprise.

## 1. Introduction

Clinical research, whether interventional ( *e.g.* , clinical trials) or observational ( *e.g.* , quasi-experimental studies, outcomes research), encompasses research conducted on humans or on materials of human origin ( *e.g.* , real-world data), and is critical to advancing medical science and public health. Conducting such research is a complex, information-intensive effort, involving multiple actors, workflows, processes, and resources. In this context emerged over 15 years ago the field of Clinical Research Informatics (CRI), a biomedical informatics sub-discipline focused on clinical research that was first defined in 2009 [ 1 ]. More recently, several literature reviews have been conducted to further frame and refine the topics of CRI in general [ 2 , 3 ]

The focus of this review is to provide an updated practical and informative overview of the CRI field based on examples of impactful papers selected during the last decade through two regular review processes: the annual “CRI year-in-review” presented by at the Informatics Summit of the American Medical Informatics Association (AMIA year-in-review), and the periodic CRI reviews in the IMIA Yearbook of Medical Informatics (IMIA Yearbook).

Our objectives are threefold:

- to provide an overview of the evolving scope and categories of focus for this subspecialty of biomedical informatics over the past 10 years;
- to highlight major contributions to the CRI field during the last decade in each category and identify efforts by the CRI community to address practical informatics needs for advancing clinical research;
- to provide insights on more recent ( *i.e.* , past two years) contributions in CRI and identify current research trends and perspectives for the CRI field.

In the sections that follow, we first describe source selection and information extraction. We then highlight major contributions according to the three dimensions presented above (CRI definition, major CRI contributions, and highlight recent research trends and perspectives).

## 2. Methods

The authors adopted a modified thematic review approach based, in a pragmatic way, on the analysis of the literature sources resulting from two review processes (AMIA CRI year-in-review/IMIA Yearbook) conducted annually since 2013.

### 2.1. Research question and scoping questions

The core research question of this review focuses on practically relevant aspects of CRI supporting various steps of the life cycle of clinical research. The authors agreed to consider the following scoping questions: “How has the definition and scope of CRI evolved along time?”; “What are examples of CRI tools and methods that have reached sufficient maturity to support clinical research?”; and “What are the current trends and major challenges for CRI research today?”

### 2.2. Information sources, search strategy and paper selection

**Figure 1.**
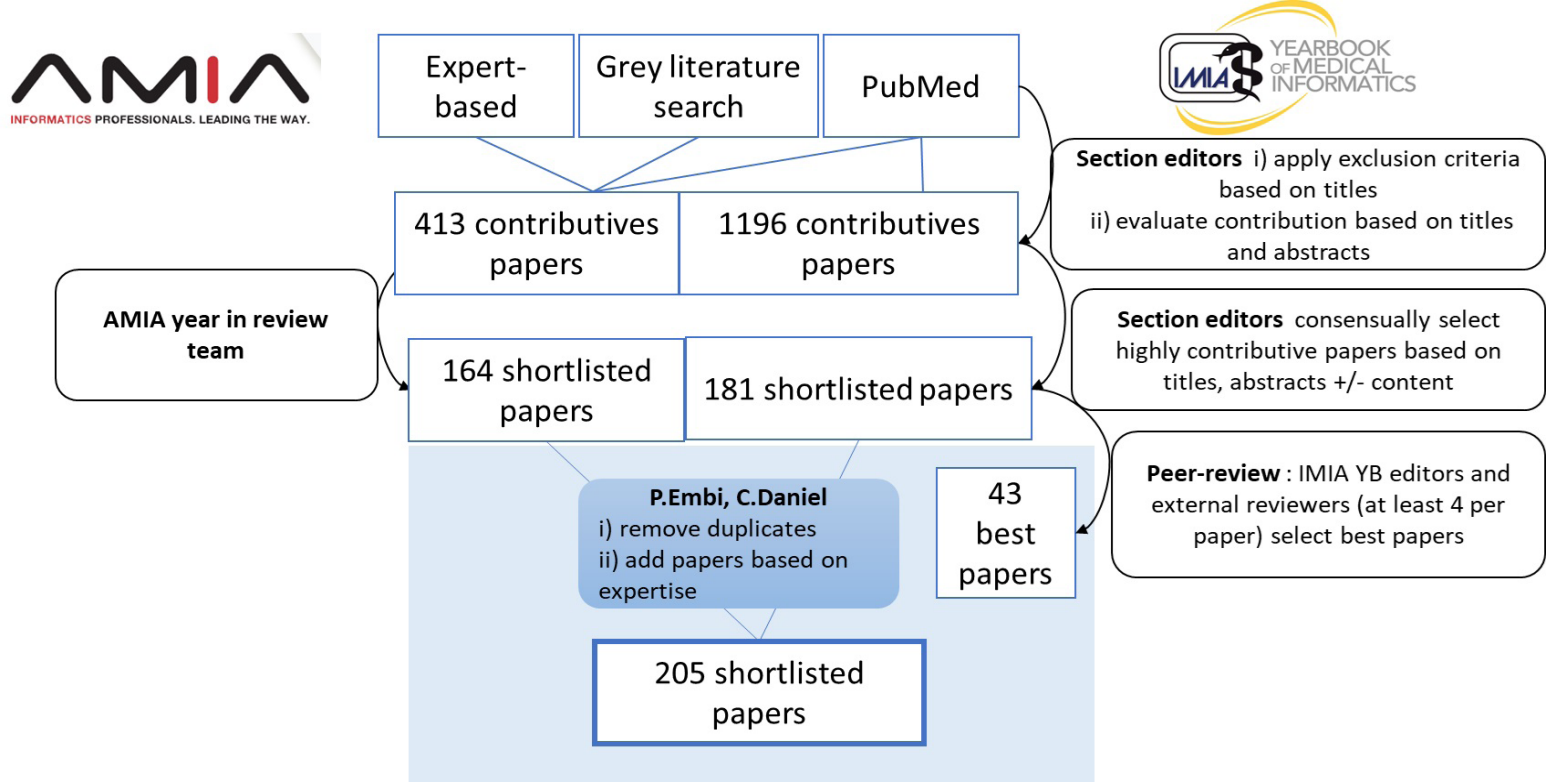
Overview of the article selection in both AMIA CRI year-in-review and IMIA Yearbook processes, and as applied for purposes of this review (highlighted in the blue box).

Table 1 summarizes the search strategies used by: (a) Embi in preparation of the “CRI year in review” presented annually at the AMIA Informatics Summit, and (b) by the editors of the CRI section of the IMIA Yearbook. Figure 1 summarizes how the authors identified and evaluated the literature sources shortlisted by both AMIA year-in-review and IMIA Yearbook processes, and then applied those approaches to this review.

**Table 1.** Search strategy and paper selection in both AMIA CRI year-in-review and IMIA Yearbook processes.

**1 – Search strategy**	
MEDLINE query via PubMed	
(a) AMIA CRI year-in-review	(b) IMIA Yearbook CRI section
(a) Initial search by MeSH terms and keywords, including:“Biomedical Research”[MeSH] AND “Informatics”[MeSH] NOT (“computational biology”[MeSH] OR “genetics”[MeSH]) (b) Limit to (e.g., English, with Abstracts, etc.) Additional articles identified using date restrictions and keyword searches with terms such as: Clinical Trials, Clinical Research, Informatics, Translational, Data Warehouse, Research Registries, Recruitment. Recommendations from colleagues.	Two conceptual axes: clinical research and informatics
	((Biomedical Research[MAJR] OR „Biomedical Research“[TIAB] OR „Clinical research“[TIAB] OR „Medical research“[TIAB] OR Nursing Research[MAJR] OR „Nursing Research“[TIAB] OR „Pharmacovigilance“[TIAB] OR „Patient Selection“[TIAB] OR „phenotyping“[TIAB] OR „genotype-phenotype associations“[TIAB] OR Epidemiologic Research Design[MAJR] OR „Epidemiological Monitoring“[TIAB] OR Evaluation Studies as Topic[MAJR] OR Clinical Studies as Topic[MAJR] OR Multicenter Studies as Topic[MAJR] OR Big data [MAJR] OR „Feasibility Studies“[TIAB] OR “clinical research informatics” [TIAB] OR “eligibility criteria” [TIAB] OR “feasibility criteria” [TIAB] OR “cohort selection” [TIAB] OR “patient recruitment” [TIAB] OR “clinical trial eligibility screening” [TIAB] OR “eligibility determination” [TIAB] OR “patient-trial matching” [TIAB] OR “protocol feasibility” [TIAB] OR “real world evidence” [TIAB] OR data science[MAJR] OR „data science“[TIAB] OR Clinical trial protocols as topic[MAJR])
	AND (medical informatics[MAJR] OR „medical informatics“[TIAB] OR „clinical informatics“[TIAB] OR „medical computer science“[TIAB] OR „medical information science“[TIAB] OR Informatics[MAJR] OR „Informatics“[TIAB] OR „electronic healthcare record“[all fields] OR „electronic health record“[all fields] OR „electronic medical record“[all fields] OR „electronic patient record“[all fields] OR „personal health record“[all fields] OR “big data“[TIAB] OR “real world data” [TIAB] OR (Data Warehousing[MeSH] OR „Data Warehousing“[TIAB] OR „Data Warehouse“[TIAB] OR big data[MeSH] OR „big data“[TIAB]))
	AND (yyyy[DP]) AND English[lang] AND hasabstract[text]
2 – Paper selection	
From the total identified, Embi selected a sampling of impactful papers to be presented in detail, and others to receive brief mentions.	Section editors apply exclusion criteria to the original set of retrieved references to select papers in the scope of CRI and blindly evaluate their contribution based on titles and abstracts. The resulting highly contributive papers are reviewed jointly by the section editors to select a consensual list of approximately 15 candidate best papers representative of all CRI categories. In conformance with the IMIA Yearbook process, these shortlisted papers are peer-reviewed by four IMIA Yearbook editors (the two section editors, and two editors in chief), and external reviewers in order to finally select four papers as best papers.

### 2.3. Final paper selection and analysis

The search strategy and paper selection described in the previous section resulted in a total of 345 (164 from the AMIA year-in-review, and 181 from the IMIA Yearbook) scientific publications, and of 327 after removal of duplicates (n=18). After additional literature sources according to the author's expertise and final selection, 205 documents remained on the shortlist included as references for this review paper. Consensus between the two reviewers, who are also the co-authors, was reached by discussion. As with the AMIA and IMIA processes on which this approach was based, the ultimate selection of impactful papers reflects the views of the authors, and is not meant an exhaustive or systematic review *per se* .

## 3. Results

### 3.1. CRI definition and categories

#### 3.1.1. CRI definition and scope

**Figure 2.**
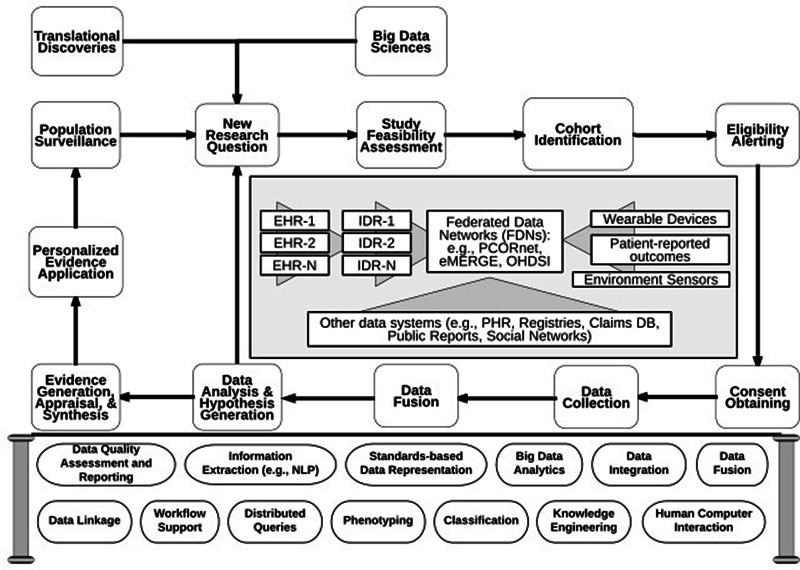
Clinical Research Informatics (CRI) conceptual framework [ 2 ].

In 2009, Embi and Payne proposed a definition of clinical research informatics (CRI) as “the subdomain of biomedical informatics concerned with the development, application, and evaluation of theories, methods, and systems to optimize the design and conduct of clinical research and the analysis, interpretation, and dissemination of the information generated” [ 1 , 4 ] Defined as the intersection of the field of clinical research and the field of biomedical informatics, CRI focuses on the development and evaluation of tools and methods to support researchers in clinical research activities including study design, patient recruitment, data collection, integration and analysis. This is further illustrated in the conceptual framework of CRI proposed by Weng and Kahn ( Figure 2 ) [ 2 ]

Subsequently, Johnson proposed a search strategy using two conceptual axes defining CRI and sharpening the boundaries with closely related fields such as computational statistics and patient care informatics [ 3 ]

As noted by these authors and others, CRI approaches and capabilities have the potential to play a prominent role in supporting widespread advances in medicine, healthcare, and public health [ 5 ]

#### 3.1.2. Methodological pillars and categories

Progress by the community of CRI researchers and developers can be depicted as advancing along major pillars of the informatics sub-discipline such as data integration, information extraction, data linkage, data quality assessment, phenotyping, data and text mining, knowledge engineering, and health technology development and assessment (workflow support, human computer interaction, evaluation) in order to reinforce methods and tools supporting the different stages. The papers selected by the AMIA year-in-review and IMIA Yearbook processes have been classified into other sub-categories related to topics and/or steps of the clinical research lifecycle for which they provide solutions [ 3 ] . Table 2 summarizes the classification of the final list of selected papers identified via the combined review processes utilized for the current review.

**Table 2.** Categorization and sub-categorization of the 205 selected papers.

Categories (Acronym, label, number of papers)			Sub categories	Corresponding stage of the clinical research study life cycle	Sources
SD-PR-EXE	Study design & executionPatient recruitment	n=43	Study Design (new CT designs)	CRI step 1: Analyzing Study Designs, feasibility studies	[ 6 7 8 9 10 11 12 13 14 15 16 17 18 19 20 21 22 23 24 25 26 27 28 29 30 31 32 33 34 35 36 37 38 39 40 41 42 43 44 45 46 47 48 ]
			Patient Recruitment	CRI step 2: Getting Participants into Studies	
			Study Execution (CRI methods & systems, collaborative workflow systems, usability and needs)	CRI step 3: Executing Studies	
PTF	Large scale research platforms	n=12	Study Execution (large scale collaborative platforms, data networks)		[ 49 50 51 52 53 54 55 56 57 58 59 60 ]
DM	Study data management	n=59	Managing Study Data (focus real-world data/phenotyping) (n=17)	CRI step 4: Managing Study Data	[ 61 62 63 64 65 66 67 68 69 70 71 72 73 74 75 76 77 ]
			Managing Study Data (focus semantic interoperability, data integration and standardization) (n=19)		[ 78 79 80 81 82 83 84 85 86 87 88 89 90 91 92 93 94 95 96 ]
			Managing Study Data (focus data quality assessment) (n=14)		[ 97 98 99 100 101 102 103 104 105 106 107 108 109 110 ]
			Managing Study Data (focus security, confidentiality) (n=9)		[ 111 112 113 114 115 116 117 118 119 ]
MKD	Data/text mining	n=18	Using Study Data (data or text mining, data visualization)	CRI step 5: Using Study Data	[ 120 121 122 123 124 125 126 127 128 129 130 131 132 133 134 135 136 137 ]
COM-PUB	Results communication and publication	n=5	Communicating Study Results (Research results dissemination)	CRI step 6: Communicating Study Results	[ 138 139 140 141 ]
			Analyzing Study Publications (knowledge representation, management, or engineering)	CRI step 7: Analyzing Study Publications	[ 142 ]
INFRA	Technical infrastructure	n=7	Architecture (Big Data, Cloud computing)		[ 143 144 145 146 147 148 149 ]
ETH-REG	Ethical & legal issues	n=11	Ethical issues		[ 150 151 152 153 154 155 156 157 158 159 160 ]
			Legal, regulatory issues		
SOC	Societal issues	n=27	Societal issues (focus governance, stakeholder participation, engagement)		[ 161 162 163 164 165 166 167 168 169 170 171 172 173 174 175 176 177 178 179 180 181 182 183 184 185 186 187 ]
			Societal issues (focus patient participation, health literacy, patient consenting)		
			Societal issues (focus business model)		
			Societal issues (focus workforce, education and training)		
LHS-SURV	Learning Health Systems Surveillance	n=10	Learning Health Systems, disease, drug & device surveillance		[ 188 189 190 191 192 193 194 195 196 197 ]
TRE	Trends	n=13	Trends		[ 1 2 3 4 5 , 198 199 200 201 202 203 204 205 ]

**Figure 3.b:**
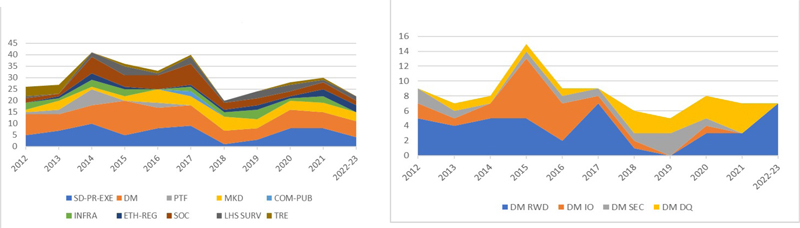
**a** . Number of papers shortlisted through both AMIA and IMIA review processes (n=327) divided in the ten main categories over time. . Number of selected/shortlisted papers in the four sub-categories of data management over time (n=90).

When the literature is plotted by categories over time (as in Figure 3 ), a different visualization of the evolution of the CRI field is highlighted, with different categories and topics varying by year.

### 3.2. Main CRI contributions supporting the life cycle of clinical research (2013-2022)

Based on our combined analysis of CRI articles selected over time by the approaches described above, this section provides an overview from 10 years of publications demonstrating progress in the multifaceted aspects of CRI supporting research and innovation in healthcare and biomedicine. Each of the sections that follow cover major categories with exemplars highlighted within.

#### 3.2.1. Study design, participant recruitment and study execution

To support the increasing complexities of clinical research over time, a number of initiatives, including the Human Studies Database [ 15 ], the database for aggregate analysis of ClinicalTrials.gov (AACT) [ 7 ], the more recent knowledge base of clinical trial eligibility criteria [ 36 ], and others [ 21 , 35 ] proposed access to clinical trials repositories allowing significant information to be leveraged and even re-used across studies. In addition, initiatives emerged that were aimed at optimizing clinical research deliverables while meeting the requirements enunciated by the U.S. Food and Drug Administration for electronic systems [ 8 ]. Major contributions over the last decade also focused on so-called *secondary use* of health data collected via routine care for optimizing clinical trials [ 6 , 9 , 22 , 28 ]. Indeed, many projects and initiatives have explored the value of EHR data for clinical trial design, patient recruitment [ 12 13 14 , 17 , 19 , 25 , 26 , 30 , 31 , 33 , 34 , 37 , 43 , 180 ], or for accelerating data collection [ 11 , 39 40 41 42 ]. Facilitated by the generalization of clinical data warehouses, some initiatives were carried out on a large scale within European projects such as the EHR4CR [ 10 , 16 , 20 , 23 , 24 , 29 ] or TRANSFoRm [ 27 ] projects. The EHR4CR project contributed to the development of the InSite commercial solution (formerly Custodix, Belgium, currently part of TriNetX) which reached the evaluation phase [ 32 ].

While randomized controlled trials (RCTs) are the gold standard for estimating treatment effects in medical research, there has also been growing interest in and use using real-world data (RWD) for use-cases including drug development, outcomes research, and observational studies. One such use case is the construction of external control arms for evaluation of efficacy in single-arm trials, particularly in cases where randomization is either infeasible or unethical [ 44 , 46 , 47 ]. In close alignment with a recently released draft framework by the Medical Device Innovation Consortium on real-world clinical evidence and *in vitro* diagnostics, publications report the possibility of substantially reducing the length and cost of diagnostic test evaluation for diseases with low prevalence to support regulatory decision-making by leveraging subjects from a RWD source [ 38 ]. The past decade has also seen the explosion of observational research using EHR data with CRI publications better illuminating our understanding of the specific caveats and considerations/guidelines for conducting observational studies and comparative effectiveness with RWD, while limiting biases [ 18 ].

#### 3.2.2. Data management

##### Real-world data and Clinical Data warehouses

Clinical data warehouses (CDW), built early on at the level of healthcare facilities (Enterprise Data Warehouses) enabled evidence generation based on real-world data to become a reality during the last decade [ 63 , 64 , 67 ]. In the United States the CDW projects, supported by the Clinical and Translational Science Award (CTSA) funding program of the National Institutes of Health (NIH), have seen increasingly high levels of maturity [ 62 , 75 ]. One successful example is the Informatics for Integrating Biology and the Bedside (i2b2) solution that has been widely used by academic health centers in the U.S. and internationally. Notable i2b2 use-cases include discovery research, case-control studies [ 66 ], enable cost-effective genome-wide studies, identify important risks from commonly used medications [ 61 ], capture and evaluate patient reported outcomes [ 48 ], access image from clinical archive [ 69 ], and enable data sharing at scale [ 70 , 79 , 92 ]. Hospital-based CDWs and multi-site data-sharing activities have also emerged in Europe and Asia over the past decade [ 65 , 71 , 72 , 76 ].

##### Interoperability and standards

Interoperability is another major pilar of CRI that has evolved. In the domain of clinical research, the Clinical Data Information Standards Committee (CDISC) has developed a number of standards for study design, data collection and analysis, and submission to the regulatory bodies [ 91 ]. The Operational Data Model (ODM) has been updated to enable the implementation of case report forms supporting more efficiently the complete clinical study lifecycle from the design phase to the collection of patient level data [ 86 ]. The complexity and heterogeneity of healthcare data collection and storage approaches has remained a motivating challenge, and there has been movement in the CRI domain toward certain common data standards.

Several research networks focused on cross-institutional data analyzes at scale developed so-called “common data models” (CDM) that require each participating system to transform their underlining data model to the relevant CDM. Examples include the OMOP CDM from the OHDSI consortium [ 89 , 94 , 120 ], the FDA Mini Sentinel's CDM, I2B2-SHRINE [ 79 ], STRIDE or SHARPn [ 81 , 143 ]. New experience has been gained from these projects and the resulting models have been analyzed and compared [ 88 ]. While some require transfer of data to a centralized solution, another approach involves federated data and metadata management. One example is the ISO/IEC 11179 standard, that has been demonstrated to semantically link disparate common data elements defined by different organizations [ 80 ]. Minimum data sets based on common data elements have been built for different disease areas [ 56 , 83 , 93 ].

Furthermore, some initiatives have been conducted to bridge standards used in clinical care with clinical research standards, often developed through parallel, and often disparate, efforts. Other harmonization efforts have been conducted within the SHARPn project in mapping Healthcare Clinical Element Models (CEMs) adopted by the Office of the National Coordinator (ONC) to data elements extracted from the CDISC templates [ 85 ]. More recently, relying on the increasing adoption of HL7's Fast Healthcare Interoperability Resources (FHIR) by the healthcare industry, many studies have reported the interest of FHIR integration profiles to facilitate the integration of care and research activities [ 39 , 92 , 95 ]. The HL7 Vulcan acceleration program 1 develops FHIR resources needed to execute prioritized use cases of secondary use of real-world data and especially EHR data. While existing information model standards ( *e.g.* , ISO 13606, openEHR, HL7 FHIR, and CDISC) define the basic semantics of health data, semantic interoperability are enabled by medical terminologies, nomenclatures, and ontologies. It is the use of these terminologies through explicitly defined value sets (terminology binding) that brings unambiguous semantics to health data. Consequently, it remains challenging to implement the shift from the “syntactic” level to the “semantic” level of data exchange enabling smart services and applications supporting communication among clinicians, researchers, and healthcare providers. In this context, Semantic Web technologies provide solutions for managing and sharing knowledge and for making the health data FAIR (Findable, Accessible, Interoperable, and Reusable) [ 87 ]. In contrast to other major foundational models for clinical research informatics ( *e.g.* , BRIDG, CDISC ODM/SDTM, HL7 FHIR, OMOP CDM), the Ontology of Clinical Research (OCRe) takes a logic-oriented ontological modeling approach and attempt to model, not only operational and administrative needs, but also study validity, confounding, and bias needed for assessing study design strength [ 15 ]. Beyond the design of ontologies in healthcare ( *e.g.* , for epilepsy [ 78 ]), research in Semantic Web technologies also includes activities contributing to CRI such as developing efficient ontology editors, and integrating ontologies into healthcare information systems [ 82 , 84 , 90 , 96 ]

##### Data quality assessment

Data quality (DQ) is essential to many CRI activities. Identifying the need for common, standardized approaches to DQ, Kahn *et al.* were motivated to initiate a community-based effort in collaboration with the Electronic Data Methods Forum (EDM) to harmonize terminologies in CRI [ 99 ]. This DQ framework has been extended beyond intrinsic aspects to cover technical, and contextual categories across the data life cycle enabling the assessment and management of RWD repositories to ensure fitness for purpose. An ontology for characterizing DQ for secondary use of EHR data has been proposed [ 98 ]. An increasing number of studies describe DQ assessment methods or tools measuring completeness of data items and other DQ dimensions [ 100 , 107 , 109 , 110 ]. Two recent reviews summarize health DQ issues and assessment practices [ 104 , 108 ]. Some initiatives involve patients to assess the quality of their clinical data [ 103 ]. Medical centers [ 102 ] or large-scale research platforms [ 101 , 106 ] are setting up continuous DQ evaluation and improvement programs.

With the increasing use of observational, non-experimental data for various purposes, the potential for introducing biases and confounding factors that are often hidden in the data must be addressed or at least carefully considered, such as when conducting RWD studies or developing and validating predictive models [ 74 , 97 , 105 ]. Recent retractions of articles published in high-profile journals reporting COVID-19 studies based on EHR demonstrate that conforming to best practices in developing robust research based on RWD is critical to promote and foster rigor, quality, and reliability of this rapidly growing field [ 73 , 140 ].

##### Security and data privacy-enhancing techniques

New research opportunities as well as artificial intelligence and machine learning techniques raise the need for greater data access but also bring new potential risks to privacy [ 113 ]. The evaluation of this risk in releasing datasets is a major concern [ 111 , 112 , 114 ]. Many studies focus on de-identification methods and privacy-enhancing techniques [ 111 , 115 , 119 ], including homomorphic encryption techniques enabling federated analysis of RWD while complying with data protection requirements [ 117 ]. An increasing number of papers propose methods for generating synthetic health data [ 116 , 118 ].

##### Large scale research platforms

Many informatics platforms enabling collaborative research using multi-institutional distributed heterogenous data continued to emerge during the last decade [ 49 ]. Large-scale data-sharing initiatives are developing at national level. As examples, in the U.S. the SHARPn project that has proposed a scalable and standards-driven infrastructure for secondary use of EHR data [ 143 ]. SHRINE implementations have been used for nationally scalable multi-site disease studies of autism co-morbidity, juvenile idiopathic arthritis, peripartum cardiomyopathy, colorectal cancer, diabetes, and others [ 79 ]. The PCORnet initiative links multiple sub-networks via adherence to a common data model. Taken together, PCORnet sites include clinical data from a cohort of 80 million patients and enable rapid access to patient populations for pragmatic clinical trials, epidemiological research, and patient-centered research on both common presentations and rare diseases [ 53 , 58 ].

In Europe, the Health Data Hub in France and the Medical Informatics Initiative in Germany are major national initiatives supporting secondary uses of health data [ 72 , 77 ]. In U.K., we can cite two major initiatives, the Clinical Practice Research Datalink (CPRD) [ 51 ] and the ClinicAl disease research using LInked Bespoke studies and Electronic health Records (CALIBER) programs enabling secondary use of nationwide big data from linked electronic health records to improve outcomes in respectively primary care and cardiovascular diseases [ 57 ].

At a broad international scale, the Observational Health Data Sciences and Informatics (OHDSI) initiative installed the OMOP common data model as a *de facto* standard for observational research based on large-scale real-word data and demonstrated the value of large-scale data sharing in the analysis of drug use [ 89 ]. Finally, during the Covid19 pandemic, national Covid databases were implemented (for example in US (e.g. NC3) [ 60 ], Germany or Spain [ 93 ]) and international data-sharing platforms were set up (such as 4CE [ 149 ] or SCOR [ 59 ] projects). In addition, CRI efforts resulted in practical implementations of biobank research systems [ 50 , 54 ].

#### 3.2.3. Data/text mining, artificial intelligence/machine learning, knowledge discovery

One of the most striking findings in CRI over the last decade is the exceptionally rapid development of highly flexible, reusable tools developed to explore, visualize and analyze complex data sets and enable training of artificial intelligence (AI) models that are likely to transform medicine. Growing access to both vast data sets and advanced computational capabilities are enabling the rise of AI technologies and methods for diagnostic or therapeutic decision-support, and other approaches of relevance to CRI. Many studies report the use of machine learning in pharmacovigilance or in precision medicine to predict outcomes such as hospital admissions or in-hospital mortality for specific populations. A significant research effort has emerged to enable federated learning of predictive models from federated data sets [ 127 ]. Particular attention is also paid to methodological and organizational issues related to studies using EHR data and to propose methods for bias reduction in studies using EHR data [ 128 , 131 , 133 , 140 ].

New infrastructures and methods for data integration/fusion, record linkage, data and text mining have enabled new data discovery opportunities. Significant research efforts in the field focused on the development and validation of phenotyping methods. Rule-based phenotype definitions are collaboratively developed and evaluated within initiatives such as eMERGE [ 121 ] or PheKB 2 [ 122 ] in U.S. or the CALIBER platform 3 [ 57 ], and the phenotype health data gateway 4 in U.K. Many studies exploit the advances in natural language processing (NLP) to extract clinical information from clinical texts [ 120 , 126 , 135 ]. Others have implemented and promoted innovative approaches for high-throughput phenotyping [ 81 , 120 , 123 124 125 , 129 , 130 , 132 , 134 ].

##### Study results communication and publication

Although of undeniable interest in the generation of evidence, especially in areas where traditional clinical trials would be unethical or infeasible, RWD should be reused considering the highest research standards and updated guidelines such as the methodological reference for conducting observational studies developed by European Network of Centers for Pharmacoepidemiology and Pharmacovigilance, ENCePP () under the aegis of the EMA (European Medicines Agency) and the FDA (Food and Drug Administration) or by research communities [ 140 , 141 ]. For example, the CONSORT-AI (Consolidated Standards of Reporting Trials-Artificial Intelligence) extension and its companion statement for clinical trial protocols: SPIRIT-AI (Standard Protocol Items: Recommendations for Interventional Trials-Artificial Intelligence) from the EQUATOR network provide recommendations (checklist items) promoting transparency and completeness in reporting clinical trials for AI interventions [ 205 ]. These recommendations assist editors and peer reviewers, as well as the general readership, to understand, interpret and critically appraise the quality of clinical trial design and risk of bias in the reported outcomes. In the radiology domain, DECIDE-AI is a new reporting guideline for artificial intelligence studies in radiology. Some other specific concerns of study research communication are e.g., the increase of self-citation [ 139 ] or tracking and efficiently displaying research evidences [ 138 , 142 ].

##### Technical infrastructure

In the current era of massive-scale digitalization of data and computationally-intensive quantitative data analytics, the CRI community has developed new infrastructure capacities that can process, analyze and store petabytes of health data [ 145 , 146 , 148 ]. The management of massive, unstructured and heterogeneous data is still challenging, especially for supporting the identification of environmental exposures as determinants of physio pathological processes and providing a coherent framework for dealing with multi-scale population data including the phenome, genome, exposome, and their interconnections in the context of the One Health paradigm [ 65 , 144 , 147 ].

##### Ethical, legal and societal issues (data governance, patient engagement, sustainability)

A major lesson that the coronavirus disease 2019 (COVID-19) pandemic has taught the scientific CRI community is that healthcare institutions shall continue to strengthen their expertise in data driven evidence generation. Since new opportunities also bring new societal challenges, most of the institutions in charge of CRI and/or CDW for research put in place governance committees of varying forms in charge of setting up the policies of data requests for internal research projects and/or external data sharing agreements as well as reviewing these requests [ 162 ]. These governance bodies need to guarantee that the intensive data-driven research is conducted in respect of the law and in line with ethical principles ( *e.g.* , that data release fits the purpose of the research), especially in the context of large-scale collaborative research. Ethical and regulatory issues related to the use of large-scale linked data and new AI technologies are especially challenging and need to be addressed to respond to the current rapidly changing data-driven agenda. Researchers have to understand complicated and sometimes contradictory legal requirements inherent to big data projects and to consider ethical obligations in order to balance potential of discovery with legal and ethical considerations [ 151 , 152 , 154 , 159 , 160 ]. Ethics review committees may have to be reformed to deal with big data research and improve their oversight capacity [ 156 , 158 ] and supportive tools (eIRB systems) could be useful in this context. Some publications focus on lessons learned and recommendations related to ethical, legal and societal issues in the specific context of the covid-19 pandemics [ 157 ] or rare diseases. In Europe, the European Institute for Innovation through Health Data (i~HD) plays a strong role in many national and international collaborative projects or initiatives involving academic research groups from all over Europe collaborating with global pharmaceutical companies in supporting the ecosystem in especially addressing ethical, legal, societal issues [ 177 ]. Key national initiatives are promoting CRI [ 161 , 166 , 171 , 172 , 178 , 179 , 181 ]. Significant research efforts in the CRI field also focused on specific societal issues : patient information and consent [ 150 , 153 , 155 , 168 , 182 ], patient involvement in the design and execution of data-intensive medical research and clinical trials [ 164 , 170 , 175 , 176 , 183 ], health equity in CRI [ 180 , 184 , 186 ], sustainability [ 68 , 163 ], the role of IT vendors [ 167 , 185 ], barriers to data sharing [ 165 , 173 ] and more generally educational issues [ 169 , 174 ].

##### Learning Health System and large-scale surveillance

CRI contributions have also advanced support for the realization of a learning health system that enables healthcare systems to objectively and continuously monitor their practices, pragmatically compare the outcomes of care over time, including patient-reported outcomes, and simulate and evaluate the impacts of certain strategic decisions on organizations or the quality of care [ 191 , 192 , 194 , 196 ]. Major research efforts also focused on data-driven approaches for signal detection in pharmacovigilance especially in post marketing drug and vaccine safety surveillance and more generally patient safety [ 188 189 190 , 193 , 195 ].

### 3.3. Recent (last two years) research trends and perspectives (2022-2023)

#### 3.3.1. Health technology assessment, maturity of CRI services and innovative clinical trial designs

Despite considerable progress over the past 10 plus years, certain challenges remain a focus for CRI now and into the future. CRI interventions still appear too often as anecdotes or quasi-experimental studies, whereas methodologically rigorous studies ( *e.g.* , randomized, controlled studies) of CRI interventions remain rare. As the field matures, more efforts will be needed to demonstrate the successes and value of CRI interventions. Moreover, there is a need to enhance the level of formalization of CRI services in large academic centers. ***Ensuring high-quality research*** relies on multiple factors including variations in organizational culture of academic medical centers, retention of research workforces, efficient multi-departmental organizational collaborations (e.g., relationship between biomedical research expertise and enterprise IT), technical skills, operational efficiencies and transparent communication of standard policies, procedures and key performance indicators [ 199 ].

Another emerging concern relevant to CRI is the development, validation, and evaluation of ***AI-based healthcare technology*** . In the context of medical AI, that survived several “winters” since the 1970's, there is a need to better understand how new AI technologies will challenge current strategies for regulating and validating AI devices for medicine and research. Recent reviews focusing on the development of AI in medicine state that AI could transform clinician workflows, patient care, administrative tasks, and augment medical knowledge and decision making [ 203 , 204 ]. They also underline unsolved challenges, from issues about training ML systems to unclear accountability making AI's implementation difficult and largely ununderstood by healthcare professionals still struggling to implement AI in their daily practice. Guidelines for clinical trial protocols for interventions involving AI are available and some papers propose AI implementation frameworks and approaches for AI development, implementation, use, monitoring and governance [ 131 , 133 , 198 ].

As the regulatory environment becomes progressively receptive to utilizing real-world evidence in clinical trials, many publications focus on various ***innovative hybrid clinical trial settings*** . Of particular interest is the use of RWD to accelerate and perhaps even approximate certain traditional research approaches. Among innovative trial designs, platform trials including multiple trial arms (conducted simultaneously and/or sequentially) on different treatments under a single master protocol. The capabilities of medical centers to perform EHR-based protocol feasibility assessment, clinical site selection, and patient pre-screening are keys for successful platform trials [ 45 ]. An increasing number of initiatives, such as the TAES (TriAl Eligibility Surveillance) system, leverage existing real-world data, using artificial intelligence and standard data models, to enhance identification of patients potentially eligible for clinical trials and generate timely notifications to physicians and simultaneously decrease the burden on research teams of manual EHR review [ 43 ]. Target trial emulation can be used for eligibility determination [ 200 ] and also to assess agreement between clinical trials and analogue real-world evidence studies. Concordance in results vary depending on the agreement metric. Emulation differences, chance, and residual confounding can contribute to divergence in results [ 201 ].

#### 3.3.2. Translational research, better integration of care and research and Learning Health Systems

There is a need to promote organizational and technical interoperability between care and research domains towards the implementation of “ ***clinical trials as care option*** ”. There are an increasing number of initiatives aiming at avoiding double data entry through the integration of electronic case report forms (eCRF) systems with electronic health records (EHR) – the “ ***eSource” or “EHR2EDC” use case*** . While technical capabilities are important, organizational priorities, structure, and the site's support of clinical research functions are equally important considerations. According to a recent study assessing site readiness for eSource, only 21% of sites were using Fast Healthcare Interoperability Resources (FHIR) standards to exchange patient data with other institutions. Respondents generally gave lower readiness for change ratings to organizations that did not have a separate research information technology group and where researchers practiced in hospitals not operated by their medical schools [ 41 ]. Although a recent study demonstrated that secure, consistent, and automated end-to-end data transmission from the treating physician to the regulatory authority was feasible, the industry-wide implementation of EHR2EDC still requires policy decisions that set the framework for the use of research data based on routine care data [ 42 ]. To overcome the difficulty of integration with EHR systems due to information security and data protection restrictions, a novel architecture for a federated electronic data capture system (fEDC) has been proposed [ 202 ].

Despite recent efforts, especially those led by HL7 organization, for example the ***HL7 FHIR Vulcan accelerator*** , formal representation of multimodal and multi-level data supporting data interoperability across clinical research and care domains is still challenging. Additional efforts are required from standard development organizations (CDISC, HL7 and DICOM) to better align the standards used in care and research domain and from the vendors of clinical and biomedical research applications to implement interoperability by design in the development of their solutions within a “single source” approach. Common data models solve many challenges of standardizing electronic health records (EHR) data but are unable to semantically integrate all of the resources [ 96 ]. Moreover, semantic interoperability enabling secondary use of clinical data requires a rigorous governance process to ensure internationally the quality of the data standardization process consistently across care and research domains. Recognizing that interoperability frameworks must be continuously adapted to the user's needs and that the update can hardly be fully automatized, a clear collaborative process is needed to efficiently support the creation of new semantic resources scoped to any additional use case of biomedical research.

The persistent challenges posed by data collection based on paradigms of ***primary use of data vs. secondary use*** remain a threat to achieving high quality/value data at the source. Currently considerable resources are dedicated to secondary use of health data and to complex pipelines curating and enriching data outside of the scope of patient care. Efforts to promote improved data quality by design in clinical settings will result in higher quality data for research and evidence generation. For ethical reasons and for supporting the implementation of the ***Learning Heath System,*** ensuring the quality as well as the privacy-preserving accessibility of clinical data for primary use should be considered a priority with major impacts on secondary use and the research agenda.

#### 3.3.3. Trends related to the technological pillar

Progress is being made in ***data quality management*** systems standardizing medical data extraction and quality control methods [ 108 109 110 ]. The development of methods and tools to promote the use of ***textual data and advanced NLP methods*** in real-world observational studies is still an active research area with important contributions from such groups as the NLP Working Group at the Observational Health Data Sciences and Informatics (OHDSI) consortium[ 135 ]. Major research efforts are also devoted to ***phenotyping*** . Clinical experts and computer scientists still experience a variety of challenges when building phenotypes, including challenges to discerning key clinical events, clinical reasoning, and temporal elements [ 136 ]. Standard representations for phenotypes are increasingly being promoted to support the creation, localization and sharing of highly specific phenotype algorithms [ 96 , 137 ]. Additional research is also needed for building secure and efficient infrastructures for “Big data” management supporting, for instance, federated machine learning on distributed data sets hosted on sovereign cloud environments [ 127 ].

#### 3.3.4. Trends related to the ethical, legal and societal issues (ELSI)

Ethical, legal and societal research continues to be needed to define and ensure an ethically-guided, fair and patient-centric approach for CRI. Such work is essential to better understand and solve practical issues related to potential biases and inequities arising from the use of RWD and AI [ 186 , 187 ]. Specifically, strategies for appropriate constituent engagement are essential to ensure that solutions like AI can meaningfully benefit patients and other end users. As progress continues toward a new era of digital health driven by cloud data storage, distributed computing, and machine learning, key stakeholders must collaborate to ensure research findings and their downstream implementations are inclusive, representative and accessible for all who should benefit, and work in CRI is key to this.

## 4. Conclusions

As stated above, our goal was to provide an updated practical and informative overview of the CRI field via a scoping reviewing of CRI publications over the last decade. By leveraging two annual review processes, the “AMIA CRI year-in-review” and IMIA yearbook approach, we started with over 1,500 publications, and ultimately selected 205 impactful papers to analyze in-depth for the current review.

The review results provide an overview of (a) the evolving scope of CRI activities based on, (b) a decade of research in CRI, highlighting major evaluations in core CRI discoveries, innovations, and practical CRI methods and tools, and (c) more recent (past 2 years) trends and areas of CRI emphasis. The findings, as well as examples of enabled large scale research projects across institutions, regionally, nationally and internationally, demonstrate a continued maturation of the field. The findings also show that some important topics remain in focus, reflecting ongoing challenges in CRI that have not yet been fully addressed. They also reveal a need for improved and more rigorous evaluation of CRI solutions and socio-technical interventions. More research is also needed to better integrate the care and research workflows and to implement and realize the goals of a Learning Heath Systems. Similarly, increased focus on building and evaluating robust technical, governance, ethical and policy approaches is essential to build and operate the secure, effective, equitable and efficient infrastructures needed for improving clinical and translational research in an increasingly data-rich, interconnected, and computationally powerful environments. As the field continues to mature, the trajectory of progress and impact that CRI has had over the past decade can be expected to continue, and that should accrue benefits for all.

## Footnotes

1
http://hl7.org/fhir/uv/vulcan-rwd/#overview

2
http://phekb.org

3
https://www.caliberresearch.org/portal

4
https://phenotypes.healthdatagateway.org/
